# AcMYB266, a key regulator of the red coloration in pineapple peel: a case of subfunctionalization in tandem duplicated genes

**DOI:** 10.1093/hr/uhae116

**Published:** 2024-04-25

**Authors:** Wei Zhang, Jing Wu, Junhu He, Chaoyang Liu, Wen Yi, Jingyao Xie, Ya Wu, Tao Xie, Jun Ma, Ziqin Zhong, Mingzhe Yang, Chengjie Chen, Aiping Luan, Yehua He

**Affiliations:** Key Laboratory of Biology and Germplasm Enhancement of Horticultural Crop in South China, Ministry of Agriculture and Rural Areas, College of Horticulture, South China Agricultural University, No. 483, Wushan Road, Wushan Street, Tianhe District, Guangzhou, Guangdong, 510642, China; Key Laboratory of Biology and Germplasm Enhancement of Horticultural Crop in South China, Ministry of Agriculture and Rural Areas, College of Horticulture, South China Agricultural University, No. 483, Wushan Road, Wushan Street, Tianhe District, Guangzhou, Guangdong, 510642, China; Tropical Crops Genetic Resources Institute, Chinese Academy of Tropical Agricultural Sciences/National Key Laboratory for Tropical Crop Breeding, Yazhouwan Technology City, Sanya, Hainan, 572024, China/Laboratory of Crop Gene Resources and Germplasm Enhancement in Southern China, Ministry of Agriculture and Rual Affairs/Key Laboratory of Tropical Crops Germplasm Resources Genetic Improvement and Innovation of Hainan Province, No.4, Xueyuan Road, Longhua District, Haikou, Hainan, 571101, China; Key Laboratory of Biology and Germplasm Enhancement of Horticultural Crop in South China, Ministry of Agriculture and Rural Areas, College of Horticulture, South China Agricultural University, No. 483, Wushan Road, Wushan Street, Tianhe District, Guangzhou, Guangdong, 510642, China; Key Laboratory of Biology and Germplasm Enhancement of Horticultural Crop in South China, Ministry of Agriculture and Rural Areas, College of Horticulture, South China Agricultural University, No. 483, Wushan Road, Wushan Street, Tianhe District, Guangzhou, Guangdong, 510642, China; School of Landscape Architecture, Guangdong Eco-Engineering Polytechnic, No. 297, Guangshan 1st Road, Tianhe District, Guangzhou, Guangdong, 510520, China; Environment and plant protection institute, Chinese Academy of Tropical Agricultural Sciences, No. 4, Xueyuan Road, Longhua District, Haikou, Hainan, 571101, China; Department of Horticulture, Foshan University, No. 18, Jiangwan 1st Road, Chancheng District, Foshan, Guangdong, 528231, China; College of Landscape Architecture, Sichuan Agricultural University, No. 211, Huimin Road, Wenjiang District, Chengdu, Sichuan, 610000, China; Key Laboratory of Biology and Germplasm Enhancement of Horticultural Crop in South China, Ministry of Agriculture and Rural Areas, College of Horticulture, South China Agricultural University, No. 483, Wushan Road, Wushan Street, Tianhe District, Guangzhou, Guangdong, 510642, China; Key Laboratory of Biology and Germplasm Enhancement of Horticultural Crop in South China, Ministry of Agriculture and Rural Areas, College of Horticulture, South China Agricultural University, No. 483, Wushan Road, Wushan Street, Tianhe District, Guangzhou, Guangdong, 510642, China; Tropical Crops Genetic Resources Institute, Chinese Academy of Tropical Agricultural Sciences/National Key Laboratory for Tropical Crop Breeding, Yazhouwan Technology City, Sanya, Hainan, 572024, China/Laboratory of Crop Gene Resources and Germplasm Enhancement in Southern China, Ministry of Agriculture and Rual Affairs/Key Laboratory of Tropical Crops Germplasm Resources Genetic Improvement and Innovation of Hainan Province, No.4, Xueyuan Road, Longhua District, Haikou, Hainan, 571101, China; Tropical Crops Genetic Resources Institute, Chinese Academy of Tropical Agricultural Sciences/National Key Laboratory for Tropical Crop Breeding, Yazhouwan Technology City, Sanya, Hainan, 572024, China/Laboratory of Crop Gene Resources and Germplasm Enhancement in Southern China, Ministry of Agriculture and Rual Affairs/Key Laboratory of Tropical Crops Germplasm Resources Genetic Improvement and Innovation of Hainan Province, No.4, Xueyuan Road, Longhua District, Haikou, Hainan, 571101, China; Key Laboratory of Biology and Germplasm Enhancement of Horticultural Crop in South China, Ministry of Agriculture and Rural Areas, College of Horticulture, South China Agricultural University, No. 483, Wushan Road, Wushan Street, Tianhe District, Guangzhou, Guangdong, 510642, China

## Abstract

Red fruit peel is an attractive target for pineapple breeding. Various pineapple accessions with distinct red coloration patterns exist; however, the precise molecular mechanism accounting for these differences remains unknown, which hinders the pineapple breeding process from combining high fruit quality with red peel. In this study, we characterized a transcription factor, AcMYB266, which is preferentially expressed in pineapple peel and positively regulates anthocyanin accumulation. Transgenic pineapple, *Arabidopsis*, and tobacco plants overexpressing *AcMYB266* exhibited significant anthocyanin accumulation. Conversely, transient silencing of this gene led to decreased anthocyanin accumulation in pineapple red bracts. In-depth analysis indicated that variations of *AcMYB266* sequences in the promoter instead of the protein-coding region seem to contribute to different red coloration patterns in peels of three representative pineapple varieties. In addition, we found that *AcMYB266* was located in a cluster of four MYB genes exclusive to and conserved in *Ananas* species. Of this cluster, each was proved to regulate anthocyanin synthesis in different pineapple tissues, illustrating an interesting case of gene subfunctionalization after tandem duplication. In summary, we have characterized *AcMYB266* as a key regulator of pineapple red fruit peel and identified an MYB cluster whose members were subfunctionalized to specifically regulate the red coloration of different pineapple tissues. The present study will assist in establishing a theoretical mechanism for pineapple breeding for red fruit peel and provide an interesting case for the investigation of gene subfunctionalization in plants.

## Introduction

*Ananas* comprises approximately eight species, classified into two groups based on their fruit usage: edible (pineapple) and ornamental [1, 2]. The ripe fruits of most edible pineapples exhibit a relatively monotone coloration, with yellow fruit peels, green leaves, and red inflorescences. In contrast, some ornamental groups are rich in anthocyanins, resulting in bright red inflorescences, peels, and even the whole plant [2, 3]. *Ananas* fruit is a conglomerate fruit developed from bracts, calyx, ovary, receptacle, and total pedicel, and its peel is composed of bracts and persistent calyx (BC) (Supplementary Data Fig. S1). In most pineapple varieties, the accumulation of anthocyanins occurs exclusively in inflorescences and heart leaves during flower bud differentiation to flowering [4, 5]. A major aim of pineapple breeding programs has been to integrate the red-skinned characteristics of ornamental pineapple species into edible varieties through hybridization, seeking red-skinned edible pineapple varieties with exceptional fresh food quality [6]. However, it was frequently observed that the fresh food quality of these red-skinned hybrid offspring fell short of that exhibited by their parent edible varieties [6]. Hence, exploring the molecular mechanism regulating anthocyanin accumulation in pineapple peel will aid in the creation of high-quality edible varieties with red skin and increase their commercial and nutritional value.

Anthocyanins are glycosylated flavonoids, a group of metabolites in the phenylalanine pathway that is widely present in the plant kingdom [7]. As one of the most important water-soluble pigments in plants, anthocyanins are widely distributed in tissues and organs, including the flowers, pulp, peels, leaves, and stems, providing color to plant tissues and organs [8–12]. In addition, anthocyanins have been found to benefit human health [13]. Anthocyanins are generated from a branch of the flavonoid synthesis pathway, whose regulatory mechanism is highly conserved in many plants [14–17]. They are synthesized from phenylalanine through the flavonoid synthesis pathway, involving a total of eight synthetase genes. Among them, the phenylalanine ammonia-lyase gene (*PAL*), chalcone synthase gene (*CHS*), chalcone isomerase (*CHI*), flavonoid-3′-hydroxylase gene (*F3′H*), and flavonoid-3′5′-hydroxylase (*F3′5′H*) genes are involved in the synthesis of precursor dihydroflavonols as early biosynthetic genes (EBGs). The downstream genes for dihydroflavonol-4-reductase (*DFR*), anthocyanin synthase (*ANS*), and flavonoid glycosyltransferase (*UFGT*) are involved in anthocyanin synthesis as late biosynthetic genes (LBGs) (Supplementary Data Fig. S2) [16, 18–20].

**Figure 1:**
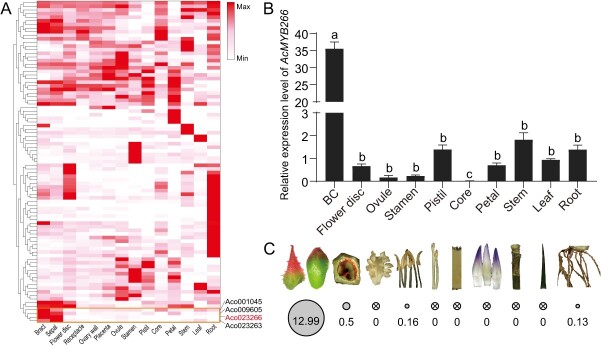
*AcMYB266* is preferentially expressed in pineapple peel. **A** Expression pattern of 89 R2R3–MYB transcription factors in different pineapple tissues. FPKM values of genes are scaled individually from 0 to 1. Red color denotes high expression levels. **B** RT–qPCR verification of *AcMYB266* expression levels in different tissues. Error bars indicate the standard deviation from three biological replicates per group. Significant differences (*P* < 0.05, *t*-test) are indicated by different letters above the columns. **C** Bubble plot visualizing expression levels of *AcMYB266* in various tissues of ‘SW’. Larger circle size denotes higher expression levels while cross lines in the circle denote undetectable expression levels.

Members of the R2R3–MYB transcription factor family are thought to be key regulators of the anthocyanin biosynthesis pathway, and bind directly to the promoters of structural genes [11, 21, 22]. In dicotyledonous plants, *AtMYB75* in *Arabidopsis thaliana* regulates anthocyanin accumulation in the hypocotyls and cotyledons of seedlings [23]. Similar findings have been reported in vegetables and fruit trees. *StMYBA1*, *StMYB113*, and *StAN1* in potatoes (*Solanum tuberosum*) promote anthocyanin accumulation in leaves or tubers [24, 25], and *MdMYB10* in apple (*Malus domestica*) is responsible for regulating fruit color [26]. Regarding monocotyledonous plants, *OsC1* in rice (*Oryza sativa*) and *TaPpm1* in wheat (*Triticum aestivum*) were confirmed to be MYB transcription factors involved in anthocyanin synthesis [27, 28]. In maize (*Zea mays*), *ZmC1* specifically regulates anthocyanin synthesis in the aleurone layers, while *ZmP1* contributes to anthocyanin regulation in other maize tissues [29, 30]. *Ananas* are monocotyledonous plants with abundant germplasm resources. The process of anthocyanin accumulation in the leaves, inflorescences, and peels of different accessions shows obvious differences, providing a variety of materials for analyzing the molecular mechanism of anthocyanin accumulation. Although a group of genes related to anthocyanin synthesis were identified through RNA-seq analyses, few transcription factors have been confirmed to regulate anthocyanin synthesis in pineapple [4, 31, 32].

In our previous studies, we obtained a spatiotemporal transcriptome profile of pineapple cultivar SW (*Ananas comosus* cv. ‘Shen Wan’) and characterized 94 R2R3–MYB family members [31, 32]. We found that a potential MYB transcription factor, *AcMYB266* (*Aco023266*; XP_020110371.1), seems to be the master regulator of the red coloration of pineapple peels. In the present study, we integrated physiological, cytological, and molecular evidence to uncover its regulatory mechanism for specifically promoting the accumulation of anthocyanins in pineapple peel and its effect on pineapple peel fading. Additionally, we examined the impact of the gene cluster that encompasses *AcMYB266* on the accumulation of anthocyanins in pineapple, as well as its evolutionary variation within and across various species.

## Results

### Identification of AcMYB266 as a potential major regulator of red coloration of pineapple fruit peels

Previously, we characterized 89 R2R3–MYB transcription factors in pineapple [31]. In-depth transcriptomic exploration in 14 pineapple tissues led to the identification of *AcMYB266 *(Fig. 1A). It was preferentially expressed in fruit peel (BC), the same as the tissue-specific accumulation pattern of anthocyanin (Fig. 1C, Supplementary Data Fig. S3). To verify these findings, we employed RT–qPCR and assessed expression levels of *AcMYB266* in 10 tissues, including BC, discs, ovules, stamens, pistils, cores, petals, stems, leaves, and roots. All results are in line with those in the transcriptomic data (Fig. 1B and C). To better characterize the function of *AcMYB266*, we cloned its complete coding sequence (CDS) which was found to be 648 nucleotides in length, encoding a 215 amino acid protein that contains an R2R3 signature domain, amino acid residues bound by bHLH, and a conserved C-terminal motif. Through a phylogenetic analysis of MYB transcription factors that regulate anthocyanin synthesis across various species, we found that AcMYB266 clusters with OsC1 and ZmC1, which regulate anthocyanin synthesis in black rice and the aleurone layers of maize (Supplementary Data Fig. S4). Localization of pineapple mesophyll protoplasts showed that AcMYB266 is a nuclear protein localized in the nucleus, conforming with the expected localization of transcription factors in cells (Supplementary Data Fig. S4).

**Figure 2:**
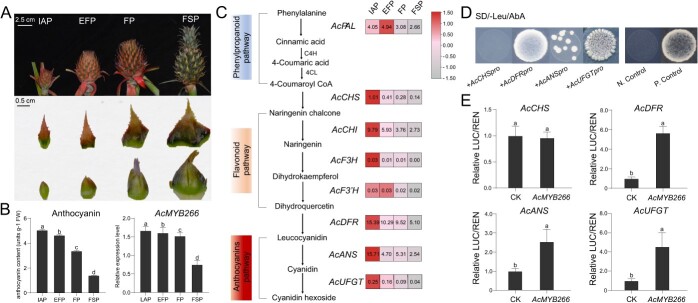
Anthocyanin content and relative expression level of *AcMYB266* during pineapple peel development and identification of *AcMYB266* target genes. **A** The inflorescence development process of SW. From left to right: IAP, EFP, FP, and FSP. The first row shows the inflorescence development process, and the second and third rows show the bracts and calyces of the four developmental stages, respectively. **B** Anthocyanin content and relative expression levels of *AcMYB266* in BC at four developmental stages. Error bars indicate standard deviation from three biological replicates per group. **C** Simplified diagram of anthocyanin synthesis pathway and heat map visualization of RT–qPCR analysis of relative expression levels of anthocyanin synthesis structural genes in pineapple in BC at four developmental stages. **D** Growth of co-transformants of *AcMYB266* and promoters of four structural genes in SD−Leu(+AbA) medium. N.Control, negative control; P.Control, positive control. **E** Dual-luciferase experiment demonstrates the activation ability of AcMYB266 on the structural gene of pineapple anthocyanin synthesis. CK, empty-effector plasmids. Error bars indicate the standard deviation from three biological replicates per group. Significant differences in **B** and **E** (*P* < 0.05, *t*-test) are indicated by different letters above the columns.

**Figure 3:**
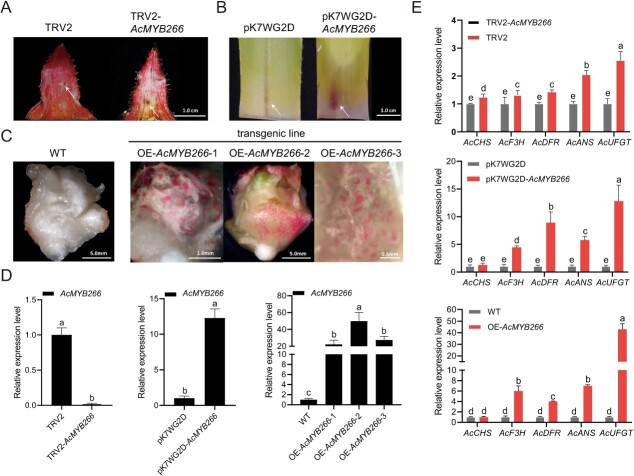
Stable or transient overexpression and silencing of *AcMYB266* affect the accumulation of anthocyanins in *Ananas*. **A** Phenotypes produced by transient silencing of *AcMYB266* in ‘Sanse’ (*A. bracteatus* var. Sanse) bracts (TRV2*-AcMYB266*). **B** Phenotypes produced by transient overexpression of *AcMYB266* in SW leaves (pK7WG2D*-AcMYB266*); **C** Phenotypes produced by pineapple callus transgenic lines stably overexpressing *AcMYB266* (OE-*AcMYB266*-1, OE-*AcMYB266*-2, OE-*AcMYB266*-3). **D** Relative expression levels of *AcMYB266* in *Ananas* materials with phenotypes shown in **A**, **B**, and **C**. Error bars indicate standard deviation from three biological replicates per group. **E** Relative expression levels of structural genes of the anthocyanin synthesis pathway in *Ananas* materials with phenotypes shown in **A**, **B**, and **C**. Error bars indicate standard deviation from three biological replicates per group. Significant differences in **D** and **E** (*P* < 0.05, *t*-test) are indicated by different letters above the columns.

### *AcMYB266* positively regulates anthocyanin accumulation in pineapple peel

To verify the association between *AcMYB266* expression and anthocyanin content, we sampled BCs at various stages of SW inflorescence development for further analysis. Samples were collected at four distinct stages (Fig. 2A): the inflorescence appearance period (IAP, 42 days after ethylene-induced flowering, following the elongation of the peduncle and independent emergence of a complete inflorescence), the early flowering period (EFP, 49 days after ethylene-induced flowering, to before floret bloom), the flowering period (FP, 60 days after ethylene-induced flowering, when florets in the middle of the inflorescence are in bloom), and the fruit set period (FSP, 70 days after ethylene-induced flowering, when all florets have withered). *AcMYB266* expression decreased during inflorescence development, with highest levels in the IAP (~2.2 times higher than FSP). This correlated with the trend in anthocyanin content found in BC (Fig. 2B). Statistical analysis showed a significant association between the two variables (Supplementary Data Fig. S5). In addition, we analyzed the expression of pineapple flavonoid synthesis genes (*AcPAL*, *AcCHI*, *AcCHS*, *AcF3H*, *AcF3′H*, *AcDFR*, *AcANS*, and *AcUFGT*) across four developmental stages. Their expression is also highest during the IAP, and their expression levels gradually decrease as the anthocyanins fade during peel development (Fig. 2C). The late biosynthetic genes *AcDFR* and *AcANS* showed higher expression, and their expression in IAP was ~1.5–500 times higher than other genes (Fig. 2C).

Whether AcMYB266 directly regulates these genes involved in the anthocyanin synthesis pathway and activates their expression would be the next question. We employed yeast one-hybrid (Y1H) experiments to confirm the effects of AcMYB266 on the early biosynthetic gene *AcCHS*, and the late biosynthetic genes *AcDFR*, *AcANS*, and *AcUFGT* of the anthocyanin synthesis pathway. After fusing the bait plasmid with the prey plasmid into Y1HGold-competent cells, we found that the co-transformed competent cells of all combinations except AcCHS grew normally on SD−Leu + AbA medium as well as the positive control (p41-plus + pGAD-53) (Fig. 2D). Thus, AcMYB266 can bind to the *AcDFR*, *AcANS*, and *AcUFGT* promoters, but not with the *AcCHS* promoter. We also conducted a dual-luciferase reporter assay to confirm the effects of AcMYB266 on the promoter activity of *AcCHS*, *AcANS*, *AcDFR*, and *AcUFGT* (Fig. 2E). Our findings revealed that the expression of AcMYB266 can activate the promoters of *AcANS*, *AcDFR*, and *AcUFGT*, but not *AcCHS*, which is consistent with the results of Y1H. The activation effects were ~2.5, 5.7, and 4.5 times greater than the control group.

We overexpressed *AcMYB266* in *Arabidopsis* and tobacco. In three transgenic *Arabidopsis* lines (OE-*AcMYB266*-At1, OE-*AcMYB266*-At2, OE-*AcMYB266*-At3) with ectopic expression levels of *AcMYB266*, red pigmentation in hypocotyls and cotyledon petioles were observed after culturing the T_3_ generation transgenic lines compared with that in the wild type (WT) *Arabidopsis* seeds on MS medium (Supplementary Data Fig. S6A). In transgenic tobacco lines overexpressing *AcMYB266*, a significant accumulation of red pigment in the stamens (anthers and filaments) of the transgenic tobacco lines was observed in the T_1_ generation compared with WT (Supplementary Data Fig. S6B). Semiquantitative PCR analysis and anthocyanin content determination showed that overexpression of *AcMYB266* enhanced anthocyanin synthesis in the transgenic *Arabidopsis* seedlings, and the stamens and petals of transgenic tobacco plants (Supplementary Data Fig. S6C and D).

As in pineapple, virus-induced gene silencing (VIGS) and transient overexpression of *AcMYB266* were conducted on red bracts and the leaf bases (which are white), respectively. Virus-induced suppression of *AcMYB266* significantly reduced anthocyanin production in red bracts, and transient overexpression of *AcMYB266* promoted the accumulation of anthocyanin in leaf bases (Fig. 3A and B). In addition, referring to our previous genetic transformation method [33], we managed to obtain transgenic pineapple plantlets overexpressing *AcMYB266*. Stable overexpression of *AcMYB266* led to distinct red pigmentation in the adventitious shoots and callus material, in contrast to the WT samples (Fig. 3C). Expression levels of *AcMYB266*, *AcCHS*, *AcF3H*, *AcDFR*, *AcANS*, and *AcUFGT* in the above transgenic lines were also assessed using RT–qPCR, which showed results consistent with the phenotypes (Fig. 3D and E).

All results confirmed that AcMYB266 is involved in the regulation of anthocyanin accumulation, and is a key activator of the pineapple anthocyanin synthesis structural genes *AcDFR*, *AcANS*, and *AcUFGT* in pineapple peels.

### Variations in the promoter region of *AcMYB266* attributed to the distinct coloration pattern of pineapple peels

**Figure 4:**
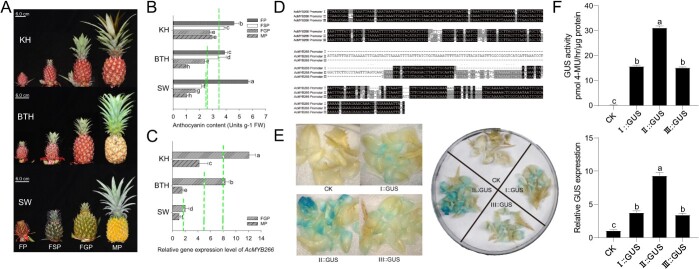
Anthocyanin accumulation types in fruit peel of representative pineapple varieties and the corresponding *AcMYB266 *promoter activation ability. **A** Comparison of the inflorescence and fruit development of three types of pineapple at four stages. The stages, from left to right, are FP (flowering period), FSP (fruit set period), FGP (fruit growth period), and MP (fruit maturity period). The types, from top to bottom, are KH germplasm, a stable accumulation type during fruiting; BTH, an unstable accumulation type during fruiting; and SW, an accumulation type during flowering. **B** Determination of the anthocyanin content in three types of pineapple at four developmental stages by measuring absorbance with a photometer. The green dashed lines in **B** represent the mean BC anthocyanin content during four developmental periods for each cultivar. Error bars indicate standard deviation from three biological replicates per group. **C** Comparison of the relative gene expression levels of *AcMYB266* in FGP and MP of three types of pineapple. The green dashed lines in **C** represent the mean expression level of *AcMYB266* during two developmental periods for each cultivar. Error bars indicate standard deviation from three biological replicates per group. **D** Three types of *AcMYB266* promoter isolated from three accessions in **A**. **E**, **F** Activity analysis of three types of *AcMYB266* promoter. **E** is the transient GUS staining of pineapple BC driven by three types of promoter of *AcMYB266*, and **F** is the analysis of GUS activity and relative expression levels in pineapple BC driven by three types of promoter of *AcMYB266*. Error bars indicate the standard deviation from three biological replicates per group. Significant differences in **B**, **C**, and **F** (*P* < 0.05, *t*-test) are indicated by different letters.

As in pineapple, different materials possess different red coloration patterns on fruit peels. We selected three representative varieties, KH (*A. comosus* cv. ‘Ka Hong’) (stable accumulation during the fruiting stage), BTH (*A. comosus* cv. ‘Red Sugar’) (unstable accumulation during the fruiting stage), and SW (accumulation during the flowering stage only) to survey how AcMYB266 is involved in this biological process (Fig. 4A–C). We cloned their full-length CDSs, which were found to be identical (Supplementary Data Fig. S7). In contrast, three types of *AcMYB266* promoter were obtained, and which are named Promoter I (from BTH), II (from KH), and III (from SW) (Fig. 4D).

Several SNPs and indels were found among these sequences and thus we hypothesize these differences in the promoter region contributed to variant activation ability. We constructed GUS expression vectors driven by three types of *AcMYB266* promoter and transiently infected pineapple fruit peel (BC). GUS staining showed that the negative control (injected with *Agrobacterium* without vector) was not stained, and the BC of II::GUS was stained the deepest. The others, transiently transformed BCs by I::GUS and III::GUS, were stained similarly. GUS gene expression and enzyme activity determination also confirmed the histochemical staining results (Fig. 4E and F), which showed that the type II promoter has the highest activity, and the activities of type I and type III are similar.

### Identification of gene cluster containing *AcMYB266* that regulates anthocyanin accumulation in various tissues of pineapple

**Figure 5:**
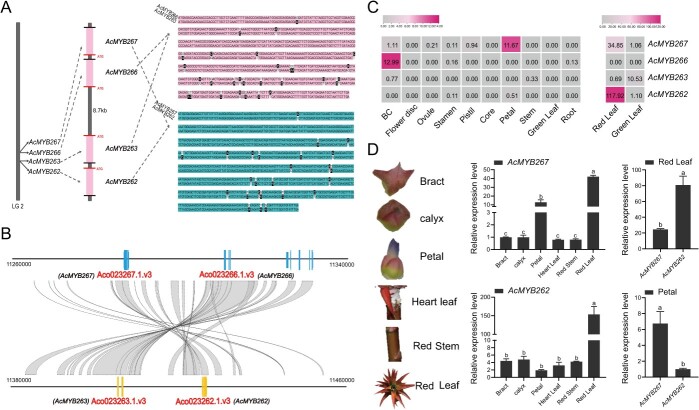
The AcMYB gene cluster and tissue-specific expression patterns of its members. **A** AcMYB gene cluster (*AcMYB267*\*AcMYB266*\*AcMYB263*\*AcMYB262*) location and member sequence analysis. **B** Collinearity analysis between AcMYB gene cluster segments. **C** Heat map visualizing the expression pattern of each gene cluster member in various tissue parts of pineapple based on RNA-seq data. **D** RT–qPCR further analyzed the expression patterns of *AcMYB267 *and *AcMYB262* in *Ananas* anthocyanin accumulation tissues. Error bars indicate the standard deviation from three biological replicates per group. Significant differences (*P* < 0.05, *t*-test) are indicated by different letters above the columns

Tandem duplication is a major driver for gene family expansion, and always results in gene members with almost identical sequences and probably redundant functions. In the present study, we happened to find that *AcMYB266* was located in an MYB gene cluster, adjacent to *AcMYB267*, *AcMYB262*, and *AcMYB263* on pineapple chromosome 2 from 1 126 000 to 11 440 000 bp (Fig. 5A). Amino acid sequence and collinear analysis showed that this cluster evolved from a tandem duplication coupled with an inverted duplication. The tandem paired genes *AcMYB267*/*AcMYB266* and *AcMYB263*/*AcMYB262* are separated by 87 kb, showing a mirrored distribution (Fig. 5A and B). These results indicate that they might have similar molecular functions. But do they take part in the same/overlapped biological process, especially the red coloration of pineapple peel that is the focus of this study? If not, what potential process they might be involved in?

To answer these questions, in addition to using the transcriptome data of 14 tissues of pineapple in our previous work [32] we performed an RNA-seq experiment on pineapple red and green leaves [which is a rare trait featured only in a few pineapple varieties, e.g. LY (*A. lucidus* var. LY), which has high anthocyanin accumulation in leaves] to more accurately describe the expression patterns of gene cluster members (Fig. 5C). Interestingly, although *AcMYB266* and *AcMYB263 *are similar in sequence (Fig. 5A), *AcMYB266* is specifically highly transcribed in pineapple BC (Fig. 5C) while *AcMYB263* is relatively highly transcribed in the red part of ‘Sanse’ leaves [4]. *AcMYB262* is specifically highly transcribed in red leaves (Fig. 5C and D). *AcMYB267*, which has a similar sequence to *AcMYB262*, is also highly transcribed in red leaves; however, it is also highly expressed in petals (Fig. 5C and D). RT–qPCR further verified the expression patterns of *AcMYB262* and *AcMYB267* in all anthocyanin-accumulating tissues of *Ananas* (LY), and the results were consistent with the transcriptome data (Fig. 5D).

Taking the results together, we found that: (i) AcMYB266 specifically regulates the accumulation of anthocyanins in the peel of pineapple; (ii) *AcMYB263* was identified as a key transcription factor gene regulating leaf anthocyanin biosynthesis in ‘Sanse’ [4]; (iii) AcMYB267 promotes anthocyanin accumulation in pineapple leaves and petals (Feng JT *et al*., data not shown; Zhang W *et al*., data not shown); (iv) *AcMYB262* is highly expressed in the red leaves of LY, and its expression levels in *F*_1_ leaves of LY (red leaves) and XG (*A. comosus* cv. ‘Xi Gua’) (green leaves) are consistent with their leaf color phenotype (Supplementary Data Fig. S8). Each member of this MYB cluster seems to implement its function in a specific tissue/stage.

### The MYB gene cluster regulating anthocyanin accumulation is unique to the genus *Ananas*

**Figure 6:**
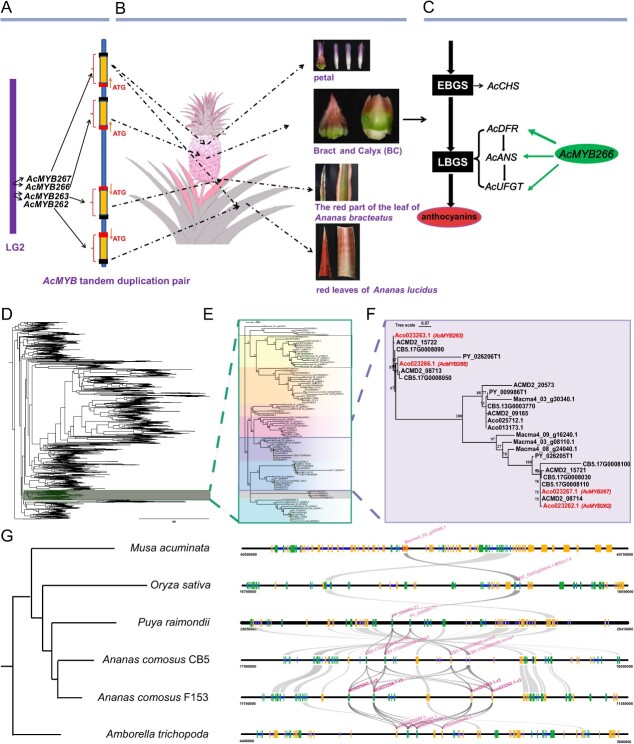
Summary of tissue-specific regulation of anthocyanin synthesis of the AARM and its exclusive conservation in *Ananas*. Distribution of the AARM cluster in *Ananas* chromosomes. Four genes are symmetrically distributed on *Ananas* chromosome 2. **B** The AAMR cluster regulates the synthesis of anthocyanin in specific tissues of *Ananas*: AcMYB267 regulates the accumulation of anthocyanin specifically in *Ananas* petals and LY leaves; AcMYB266 regulates the accumulation of anthocyanin specifically in *Ananas* peel (BC); AcMYB262 regulates the accumulation of anthocyanin specifically in LY leaves; AcMYB263 regulates the accumulation of anthocyanin specifically in ‘Sanse’ leaves. **C** Regulatory mechanism by which AcMYB266 promotes anthocyanin synthesis in pineapple peel. AcMYB266 targets the promoter sequences of pineapple anthocyanin synthesis late genes (*AcDFR*, *AcANS*, and *AcUFGT*) and activates their expression. The width of the arrows corresponds to activation intensity. EBGS, early biosynthetic gene; LBGS, late biosynthetic gene. **D**–**F** Evolutionary analysis of MYB transcription factors in *Arabidopsis*, *Amborella trichopoda*, banana, rice, CB5, F153, MD-2, and PY. **G** Species trees were generated for *Amborella trichopoda*, banana, rice, CB5, F153, and PY, and collinearity analysis was performed on MYB transcription factors that are homologous to the members of the AARM cluster in each of these regions. Green and yellow rectangles represent genes on the forward and reversed chromosome strands, respectively.

As all the above four MYB transcription factors regulate anthocyanin accumulation in pineapple (Fig. 6A–C), we hereafter named it the AARM (Anthocyanin Accumulation Related Module) cluster as it seems to be an interesting case of gene subfunctionalization after duplication. We were then interested in how and when the AARM cluster originates. Is it specific to *Ananas* or is it broadly distributed maybe in monocots or even the whole plant kingdom? We analyzed genomes of three pineapple varieties, *A. bracteatus* var. CB5, *A. comosus* cv. F153, *A. comosus* cv. MD-2, PY (*Puya raimondii*) (a sister genus of *Ananas*), rice, banana (*Musa acuminata*), *Amborella trichopoda* and *Arabidopsis* [34–40]. In total, 223, 216, 203, 183, 137, 518, 264, and 247 MYB transcription factors were found in CB5, F153, MD-2, and PY, rice, *Amborella trichopoda*, banana, and *Arabidopsis*, respectively. Combining phylogenetic and gene family analysis (Fig. 6D–F), we found that while the whole MYB transcription factor family in *Ananas* seems to be contracted compared with that in *Amborella trichopoda* (the basal angiosperm) and banana, the AARM cluster branch showed a tendency to expand.

To explore the origin of the AARM cluster, we conducted local collinearity analysis and found that the collinear block containing AARM is conserved only in *Ananas* (Fig. 6D–G). While there are four MYB family members in the associated region of the basal angiosperm *Amborella trichopoda*, there is no collinear relationship with pineapple F153 in the adjacent region, and the MYBs are also differently arranged. In both rice and banana, only one MYB gene is present, and no tandem duplication has occurred. In the association region of PY, which belongs to the same family (Bromeliaceae) but a different genus, only one pair of tandemly arranged MYBs (PY_026205T1/PY026206T1) is present, without any mirror image MYBs resulting from a similar duplication and inversion (Fig. 6G). In pineapple CB5, two pairs of tandemly arranged MYB transcription factors (CB5.17G0008030/CB5.17G0008050 and CB5.17G0008090/CB5.17G0008100) exist with opposite transcriptional directions, which is completely consistent with the distribution of AARM cluster members in F153, and they are distributed in a mirror-symmetric manner at a distance of 9 kb. The sequence identity between CB5.17G0008030 and CB5.17G0008100, CB5.17G0008050, and CB5.17G0008090 reached 96% (Fig. 6G, Supplementary Data Fig. S9). Taking these results together, the AARM cluster is supposed to have originated from a common ancestor of *Ananas* and is also exclusively conserved in *Ananas*.

## Discussion

### AcMYB266 is a key regulator of the red coloration of pineapple peel

Pineapple is an important horticultural plant, and the level of anthocyanin in the peel directly affects its commercial potential. Our recent research, which employed a multi-omics strategy, has revealed that anthocyanin plays a critical role in the development of red coloration in the peel of pineapple [41]. Until now, the molecular regulatory mechanism of anthocyanin accumulation in pineapple has been largely unknown. The present study has identified an R2R3-type transcription factor gene, *AcMYB266*, which exhibits specific expression in the peel of pineapple that accumulates anthocyanins (Fig. 1). RT–qPCR, Y1H experiments, and stable overexpression of *AcMYB266* in pineapple, *Arabidopsis*, and tobacco, as well as transient expression and silencing experiments in pineapple, collectively demonstrated its positive regulation of anthocyanin accumulation in pineapple (Fig. 3, Supplementary Data Fig. S6). This result indicated that the function of AcMYB266 in promoting anthocyanin accumulation is conserved in monocots and dicots. Three types of promoter of *AcMYB266* were obtained from representative accessions, and their activities were significantly different (Fig. 4D–F). This finding leads us to speculate about the close association between the difference in promoter sequence defined the expression levels of *AcMYB266* and thus affected the timing of anthocyanin accumulation of anthocyanin accumulation in pineapple peel.

### The AARM cluster in *Ananas* is an interesting case of subfunctionalization in tandem duplicated genes

We identified four transcription factors that individually regulate anthocyanin accumulation in different tissues of pineapple. These four genes occur as two pairs of tandem MYBs, which are gathered together in a small gene cluster (the AARM cluster) located on the LG2 chromosome of pineapple (F153) (Fig. 6A). Regional collinearity analysis among species found that the MYB genes in the AARM cluster were acquired or lost during evolution. There are MYB clusters in both *Amborella trichopoda* and bromeliads, but the tandemly replicated MYB genes in rice and banana may be lost (Fig. 6G).

Gene duplication has made a major contribution to the evolution of organisms, and these genes may be lost, subfunctionalized, or neofunctionalized following natural selection [42, 43]. A similar phenomenon exists in the AARM cluster of this study. The four transcription factors *AcMYB266*, *AcMYB263*, *AcMYB267*, and *AcMYB262* with highly homologous sequences were specifically expressed in different tissues of *Ananas* plants (Fig. 5). The inverted duplication of genes in *Ananas* gave rise to a gene cluster containing the four MYB transcription factors, and each MYB transcription factor underwent subfunctionalization. Similar findings were also found in citrus: a gene, *Ruby2*, with high homology was found near a known gene, *Ruby1*, that regulates anthocyanin accumulation, forming a cluster with *Ruby1*. They exerted different anthocyanin regulation abilities in different accessions of citrus [44]. Thus, it is plausible to assume that the AARM cluster’s four MYB transcription factors should have a common ancestor gene, *AcMYB*, which possesses the potential to regulate anthocyanin accumulation in all organs of *Ananas.*

There is also an interesting finding of MYB homologies closely related to the AARM cluster members in other species. In different species, MYB transcription factors within the same clade as the AARM cluster display a divergence in their functions and regulatory locations in tissue. The members found in rice (LOC_Os03g29614.1.MSUv7.0, ATR0598G349.1) were previously reported to regulate the accumulation of anthocyanins in the seed coat of rice [45]. However, the member in the banana (Macma4_03_g30340.1) is a TT2-like transcription factor, which has been confirmed to synthesize proanthocyanidins in *Arabidopsis* [46]. In the present study, the MYB transcription factor in the AARM cluster has been demonstrated to promote the production of anthocyanins in pineapple, whereas its expression in seed coat tissue is comparatively low (Supplementary Data Fig. S10). This suggests that the function and regulated tissue of action of the AARM cluster homologs have diverged during evolution. Subfuntionalization happens in gene orthologs within and across species. The four members of the AARM cluster regulate anthocyanin accumulation in different tissues of pineapple, which is possibly attributable to gene subfunctionalization after duplication in *Ananas*.

### Conclusions

In pineapple, limited research has been reported on the regulation of fruit peel color. Through a systematic study, we characterized *AcMYB266 *to be specifically expressed in peels and confirmed its role in regulating the accumulation of anthocyanins. The variable promoter sequence of *AcMYB266* corresponds to different gene activation abilities, and thus to the distinct red color patterns of three representative pineapple varieties. In addition, we found a cluster of four *AcMYB* genes in pineapple, whose members were connected in pairs and distributed in mirror images on chromosome 2. They seem to have undergone subfunctionalization and regulated the accumulation of anthocyanins in different tissues of pineapple. These findings will provide a basis for further exploration of the molecular mechanism of the red coloration of pineapple fruit peel, and also serve as an interesting case for the study of gene duplication and subfunctionalization in plants.

## Materials and methods

### Plant materials

Tobacco (*Nicotiana tabacum* cv. K326) and *A. thaliana* Columbia-0 were used as recipients for heterologous stable genetic transformation. The callus of *Ananas* cultivar SW (*A. comosus* cv*.* ‘Shen Wan’) was employed as the recipient for *Agrobacterium*-mediated stable genetic transformation, and the leaf tissue served as the transient overexpression material. The large-bracted *A. bracteatus* variety ‘Sanse’ was employed for VIGS transformation experiments. Tobacco (*Nicotiana benthamiana*) was used as a material for the dual-luciferase assay. The *Ananas* cultivars SW, KH (*A. comosus* cv. ‘Ka Hong’), and BTH (*A. comosus* cv. ‘Red Sugar’) were selected as representative varieties demonstrating three distinct types of anthocyanin accumulation in *Ananas* peel. LY (*A. lucidus* var. LY) and XG (*A. comosus* cv. ‘Xi Gua’) were used as parental plants to generate a total of 11 hybrid offspring. The aforementioned pineapple experimental materials were sourced from the Pineapple Germplasm Resource Garden, located at the Institute of Tropical Crops Variety Resources, Chinese Academy of Tropical Agricultural Sciences in Danzhou, Hainan province.

### Determination of anthocyanin content

One hundred milligrams of sample was dissolved in 5 ml of methanol containing 0.1% HCl, and the sample was incubated at 4°C in the dark for 24 h and oscillated three times. The supernatant was collected by centrifugation at 12 000 rpm and 4°C for 15 min, and absorbance was obtained at 530 and 650 nm using a microplate reader (Fluoroskan Ascent FL). Total anthocyanin content was calculated according to the following formula: OD_600_ = (A530 − 0.25 × A650) g^−1^. All analyses and error bars were determined from at least three biological replicates.

### Total RNA extraction and RT–qPCR analysis

Total RNA was extracted using Kangwei Century Bio-Company’s extraction kit (CW2598S), and RT–qPCR was conducted with the assistance of Yeasen’s Reverse Transcription Kit (11141ES60), quantitative reagent (11203ES08), and a Bio-Rad fluorescence quantitative PCR instrument (CFX Touch). The RT–qPCR results were normalized with reference genes (*NtActin*, *AtActin*, *AcActin*) and then the relative expression levels of genes were calculated using the 2^−ΔΔCT^method (primers are listed in Supplementary Data Table S1). All analyses and error bars were determined from at least three biological replicates.

### Gene cloning and expression vector construction

We designed sense and antisense primers (primers are listed in Supplementary Data Table S1) based on the *Ananas* genome in the NCBI database (NCBI Taxonomy ID: 4615). Leaf cDNA from SW was used as the PCR template. Amplified fragments were connected to the T-vector pCloon 007 and confirmed by Sanger sequencing before being used in subsequent experiments. Overexpression vectors were constructed by Gateway technology using the primers listed in Supplementary Data Table S1 [47]. The target fragment of *AcMYB266* was ligated into the entry vector pDONR221 in the BP recombination reaction. The recombination reaction followed kit instructions from Applied Biological Materials (ABM, Richmond, Canada). The recombinant plasmid pDONR-*AcMYB266* was then subjected to the LR recombination reaction, resulting in the 35S promoter-driven overexpression recombinant plasmid pK7WG2D-*AcMYB266*.

### Virus-induced gene silencing

VIGS was carried out as described previously [48]. The CDS of *AcMYB266* was inserted into the pTRV2 vector to generate the recombinant plasmid (pTRV2-*AcMYB266*). *Agrobacterium tumefaciens* (GV3101) colonies containing pTRV2-*AcMYB266*, pTRV2, and pTRV1 respectively were suspended in permeation buffer containing 10 mM MgCl_2_, 10 mM MES, and 150 μM acetosyringone until the OD_600_ reached 0.8. The pTRV2-*AcMYB266* and pTRV2 vectors were mixed with the pTRV1 suspension at a ratio of 1:1 and allowed to stand in the dark for 4–6 h. The mixture of pTRV1 + pTRV2-*AcMYB266* or pTRV1 + pTRV2 was injected into the middle and upper part of the bracts using an Injex-30 syringe during fruit setting. Six inflorescences were injected per injection, for a total of 240 bracts, each receiving 100 μl of the mixture. The treated inflorescences were subjected to 12 h of dark incubation, followed by cultivation under normal light conditions. The phenotype was observed after 5 days.

### Transient overexpression in pineapple leaves

The vector pK7WG2D-*AcMYB266* was transferred into *A. tumefacien**s*** GV3101 by the freeze–thaw method. Positive colonies were selected and suspended in permeation buffer containing 10 mM MgCl_2_, 10 mM MES, and 150 μM acetosyringone solution with an OD_600_ of **~**0.6. We used an Injex-30 type syringe to administer the pK7WG2D*-AcMYB266* and pK7WG2D solutions into the leaf base of SW. Each leaf was injected with 50 μl of the mixture. The leaves were treated in the dark for 12 h after injection before being cultured under normal light for 5 days**;** the phenotype was then observed and RNA was sampled.

### Generation of transgenic lines of pineapple, tobacco, and *Arabidopsis* overexpressing *AcMYB266*

The overexpression vector pK7WG2D-*AcMYB266* driven by the 35S promoter was mediated by *A. tumefaciens* GV3101 to transform pineapple, tobacco, and *Arabidopsis* plants through previous research methods [33, 49, 50]. Carbenicillin (200 mg/l) and kanamycin (50 mg/l) were used for selecting the transgenic tobacco and *Arabidopsis* lines. After rooting and acclimation, transgenic plants were transferred to the greenhouse and grown until flowering. Resistant plants were verified by RT–qPCR.

### *AcMYB266* promoter cloning and expression vector construction

DNA was extracted from leaves of KH, BTH, and SW. Based on *Ananas* genome data, the nucleotide sequence ~1000 bp upstream of the *AcMYB266* coding region was selected for promoter cloning (Supplementary Data Table S1). The obtained *AcMYB266* promoter (*AcMYB266* Promoter I, *AcMYB266* Promoter II, *AcMYB266* Promoter III) was connected to the expression vector pBI121 instead of the 35S promoter. We obtained three expression vectors driven by *AcMYB266* promoters (I::GUS, II::GUS, III::GUS).

### *AcMYB266* promoter activity analysis

The I::GUS, II::GUS, and III::GUS vectors were transformed into *A. tumefaciens* GV3101, and the infection solution was prepared as in the genetic transformation experiment with pineapple. On the day of transient infection, *Ananas* bracts and calyces (BC) in the flowering stage were collected from the base, disinfected with 75% alcohol, and then infected using the vacuum infiltration method [51]. Infection was at 101 kpa for 5 min, air pressure was reduced to 0, and then infection was repeated at 101 kpa for 5 min. Sterile filter paper was used to absorb excess water from the infected BC, which were spread on co-culture medium (MS + 2 mg/l 6-BA and 1 mg/l NAA), placed at 24°C, and cultured in the dark for 3 days. After culture, the material was washed with sterile water for 15 min, and then MS liquid culture medium containing 500 mg/l carbenicillin for 15 min. After absorbing the water, a portion was taken for GUS histochemical staining using a GUS staining kit (Beijing Coolaber Technology Co., Ltd, Beijing, China). The remaining transient transformation materials were subjected to GUS gene expression analysis and enzyme activity assay (GUS enzymatic activity assay kit, Beijing Coolaber Technology Co.**,** Ltd).

### Yeast one-hybrid assay

The complete *AcMYB266* coding sequence was cloned and inserted into the pGADT7 vector as the prey plasmid, while promoter fragments of *AcCHS*, *AcDFR*, *AcANS*, and *AcUFGT* were cloned into the pAbAi vector as the bait plasmid. The bait plasmids were transformed into the Y1H Gold strain according to the manufacturer’s instructions. The prey plasmid was transformed into a bait yeast strain, and DNA**–**protein interaction was determined by screening them on an SD medium with minimal inhibitory concentrations of aureobasidin A (AbA) and without leucine. Primers used in the relevant constructs are shown in Supplementary Data Table S1.

### Dual-luciferase reporter assay

Promoter fragments of *AcCHS*, *AcDFR*, *AcANS*, and *AcUFGT* were individually cloned into the KpnI and NcoI sites in the p0800-Luc vector [52]. The successfully constructed p0800-Luc vector and *AcMYB266* expression vector pK7WG2D-*AcMYB266* were respectively transformed into *A. tumefaciens* GV3101-pSoup and GV3101. pK7WG2D-*AcMYB266* was mixed with the promoter sequence constructs at a 1:5 (v/v) ratio and injected into 6-week-old tobacco (*N. benthamiana*) leaves. The transactivation activities ratio of firefly luciferase and *Renilla* luciferase were tested using a Dual-Luciferase Reporter Assay System (E1910, Promega, USA). Primers used in the relevant constructs are shown in Supplementary Data Table S1.

### Data sources and sequence retrieval

The genome, protein sequence, and annotation files for F153 (*A. comosus* cv. F153), rice, *Amborella trichopoda*, and *Arabidopsis* were obtained from Phytozome (http://www.phytozome.net/). The corresponding files for MD-2 (*A. comosus* cv. MD-2) were obtained from the NCBI (https://www.ncbi.nlm.nih.gov/). The corresponding files for CB5 (*A. bracteatus* var. CB5) were obtained from Ming Laboratory Developmental and Biomass Genomics (https://www.life.illinois.edu/ming/LabWebPage/Downloads.html). The corresponding files for PY (*Puya raimondii*) were obtained from the Figshare data-sharing website (https://doi.org/10.6084/m9.figshare.15015288.v1). The corresponding files for banana (*Musa* × *paradisiaca*) were obtained from the Banana Genome Hub (https://banana-genome-hub.southgreen.fr/data_search/organism).

### Identification, evolution, and collinearity analysis of MYBs

To obtain the MYB members in each species, we obtained the hidden Markov models of the MYB DNA-binding domain (PF00249) from the Pfam database (http://pfam.xfam.org/). We used HMMER3.3 (http://hmmer.org/) to query each species’ protein sequence for the MYB domain, after which the NCBI Batch CDD server was used to further check for the MYB core sequence. Complete MYB protein sequences were used to study their evolutionary relationships. MAFFT with default parameters was used for multiple sequence alignment of MYB amino acid sequences, and FastTree was used to construct the maximum likelihood tree. Species trees were manually drawn according to previous research [53]. We performed local collinearity analysis between species based on the species tree using TBtools-II [54]. To visualize the results we used the Genome Region Compare Advanced Suite function with the following parameters: CPUs for BlastP, 2; e-value, 1e−10; maxHsp, 500; minLen, 30. TBtools-II.

### Statistical analysis

All experiments were performed with at least three biological replicates, and the results were expressed as error bars. Differences were analyzed by *t*-test and were considered statistically significant when *P* < 0.05.

## Supplementary Material

Web_Material_uhae116

## Data Availability

The data underlying this article are available in the Sequence Read Archive (SRA) at NCBI under Project ID PRJNA483249 and PRJNA1094551.
