# Supplementary material for: AcMYB266, a key regulator of the red coloration in pineapple peel: a case of subfunctionalization in tandem duplicated genes
Source: Hortic Res. 2024 Apr 25;11(6):uhae116. doi: 10.1093/hr/uhae116 (PMC11197299; doi:10.1093/hr/uhae116)
Supplement: Web_Material_uhae116 [file web_material_uhae116.docx]

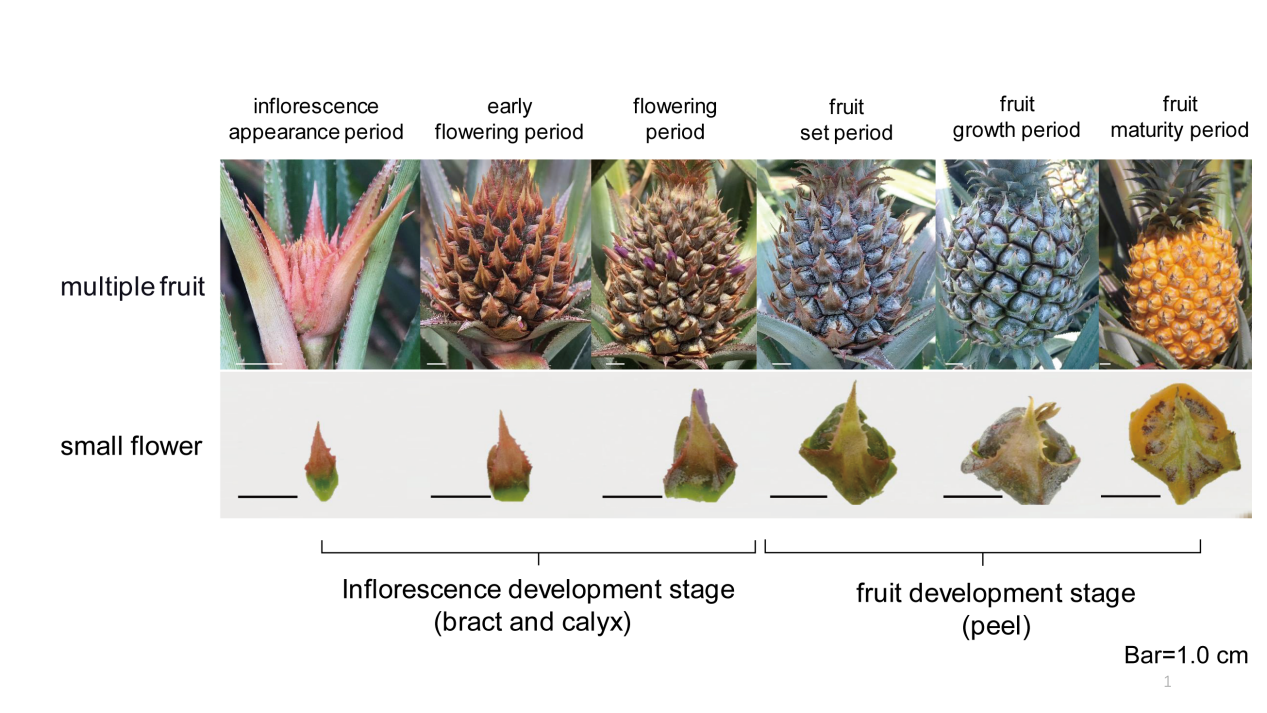


Figure S1. The process of developing pineapple bracts and calyxes (BC) into peel.

*Ananas* inflorescence and fruit development process


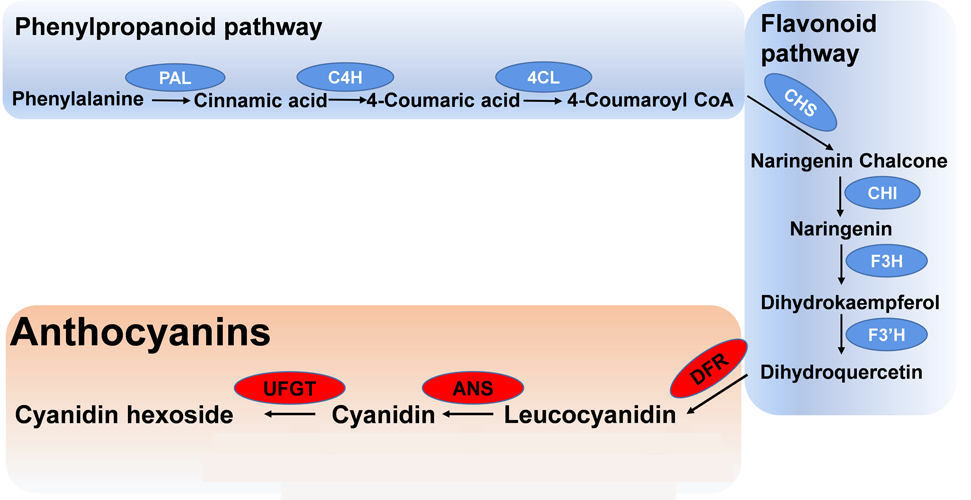


Figure S2. Plant anthocyanin synthesis pathway.

Figure showing the phenylpropanoid, flavonoid and anthocyanin pathway


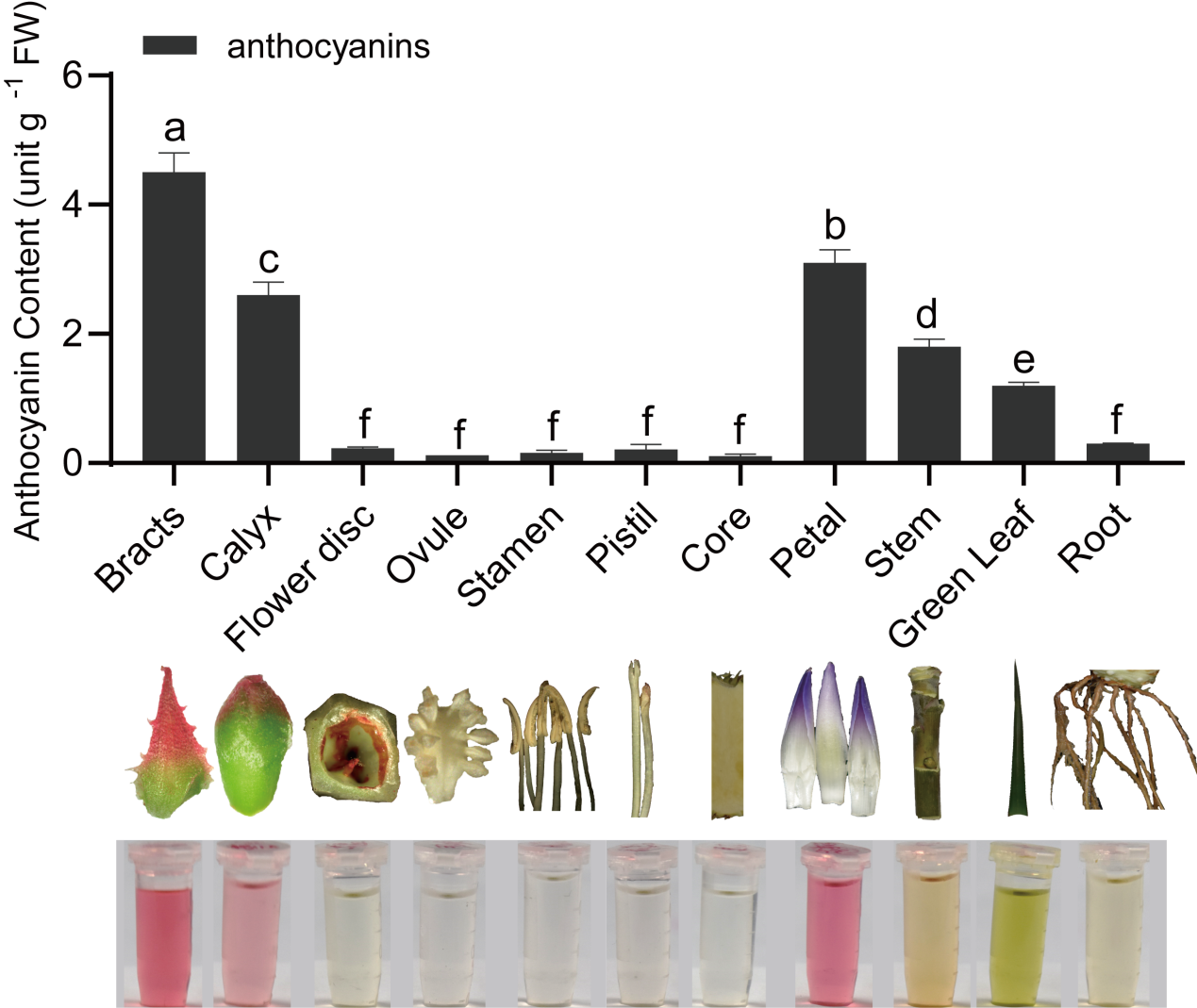


Figure S3. Anthocyanin content in various tissues of Ananas.

Anthocyanin content in 11 tissues of *Ananas* bracts, calyx, flower discs, ovules, stamens, pistils, fruit core, petals, stems, green leaves and roots


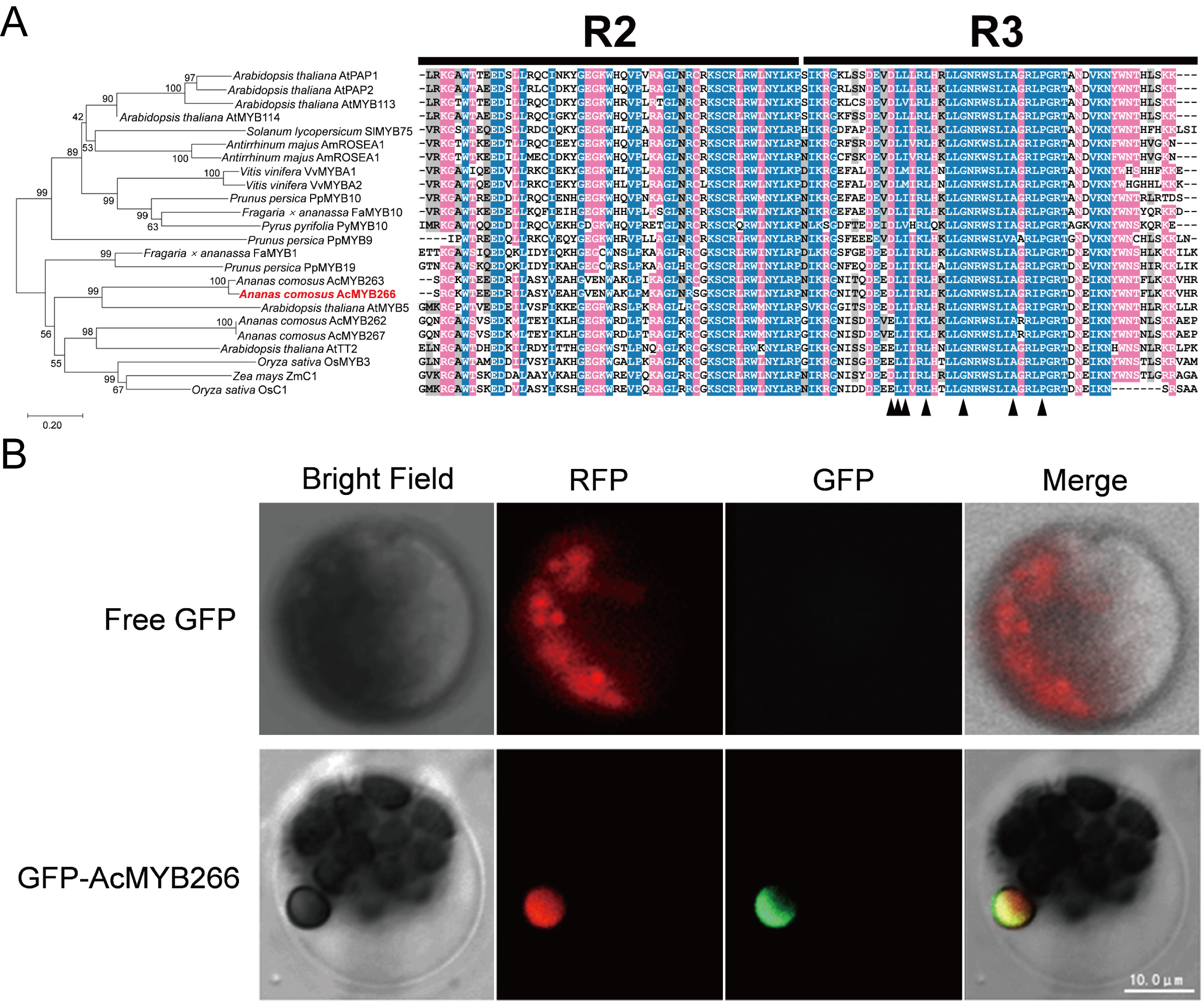


Figure S4. Molecular characterization of AcMYB266.

1. AcMYB266 sequence characterization. The left panel shows the phylogenetic analysis of AcMYB266 and other plant R2R3-MYB transcription factors. MEGA software was used to reconstruct a phylogenetic tree with the neighbor-joining method. The right part shows the alignment of the AcMYB266 protein sequences with selected homologs using GENEOC software. Amino acids with 100% identity were highlighted with a blue background, and amino acids with ≥71% similarity were shown with a pink background. The R2R3 domain is indicated by black boxes and residues required for interaction with bHLH are indicated by black arrows; B. Subcellular localization of GFP: AcMYB266, a fusion protein in *Ananas* mesophyll protoplasts. Free GFP served as a control. Bar = 10 μm.


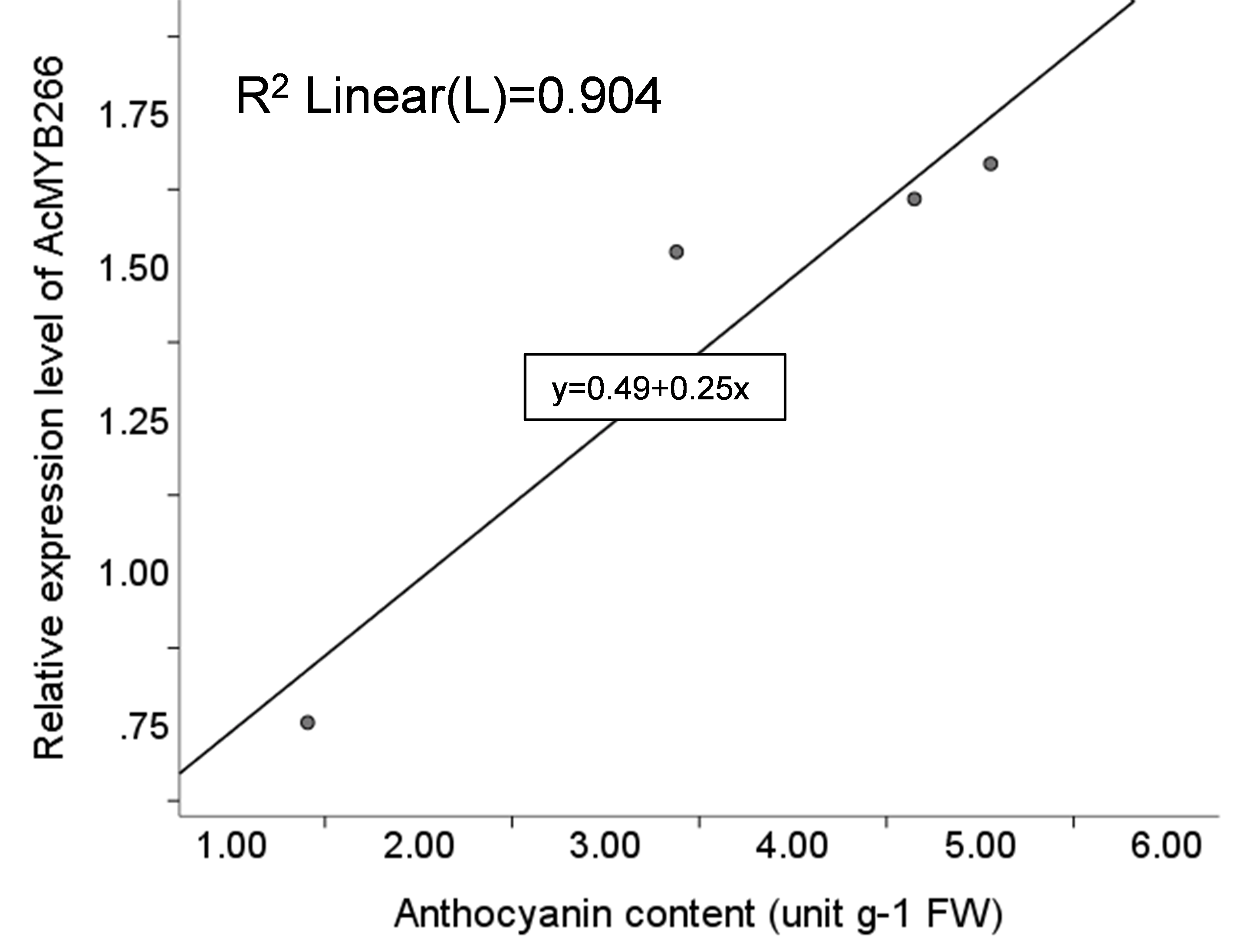


Figure S5. The scatter plot shows the correlation between BC anthocyanin content and the relative expression of *AcMYB266* in four developmental stages (IAP, EFP, FP, FSP) of "SW" inflorescence

P=0.038; r=0.95088


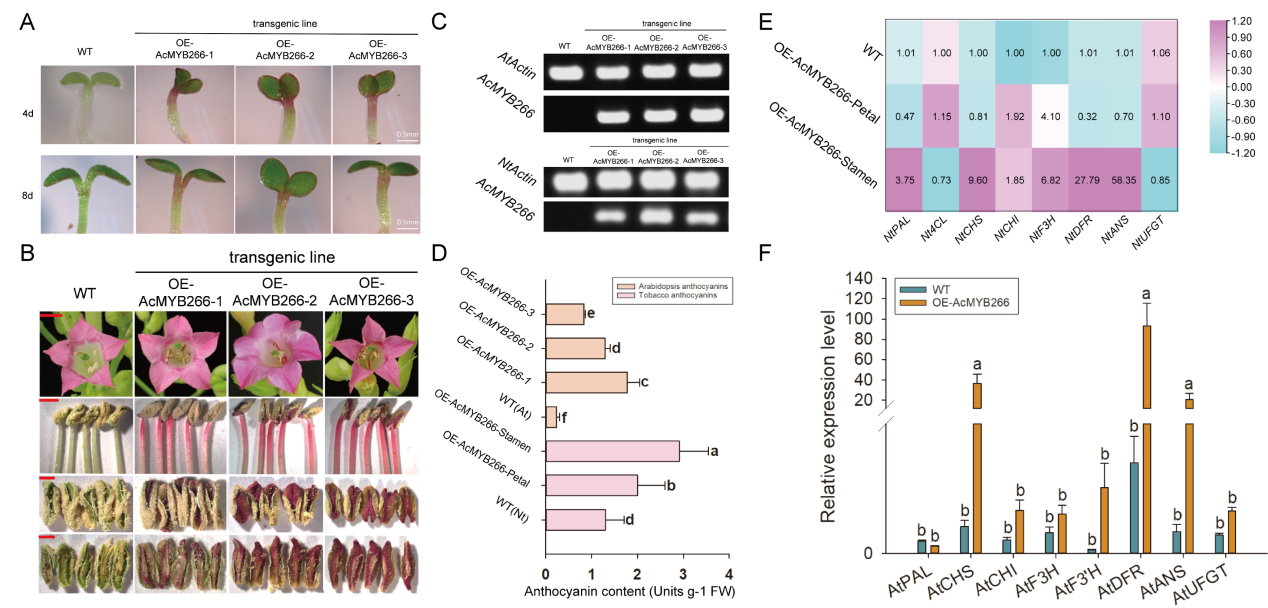


Figure S6. Effects of overexpression of AcMYB266 on anthocyanin accumulation in transgenic Arabidopsis and tobacco

1. Phenotypes of *Arabidopsis* transgenic lines (OE-*AcMYB266*-At1, OE-*AcMYB266*-At2, and OE-*AcMYB266*-At3) and WT at 4 d and 8 d of age (after sowing) on MS medium; B. Phenotypes of tobacco transgenic lines (OE-*AcMYB266*-Nt1, OE-*AcMYB266*-Nt2, and OE-*AcMYB266*-Nt3) and WT in the petal, stamen, anther, and transverse section of anther. (top to bottom); C, D. Determination of total anthocyanin content in WT *Arabidopsis* and transgenic *Arabidopsis* lines, WT tobacco, and transgenic tobacco lines, and electrophoresis analysis of *AcMYB266* transcription levels. OE-*AcMYB266*-NtPetal and OE-*AcMYB266*-NtStamen in D represent the average anthocyanin content of petals and stamens in the three transgenic tobacco lines; E, F. RT-qPCR analysis of eight *Arabidopsis* and tobacco anthocyanin synthesis structural genes in *Arabidopsis* transgenic and WT lines, and tobacco transgenic and WT lines, respectively. OE-*AcMYB266*-NtPetal and OE-*AcMYB266*-NtStamen in e represent the average relative expression levels of *AcMYB266* in petals and stamens in the three transgenic tobacco lines. F is the average relative expression level of the three transgenic lines of *Arabidopsis*.


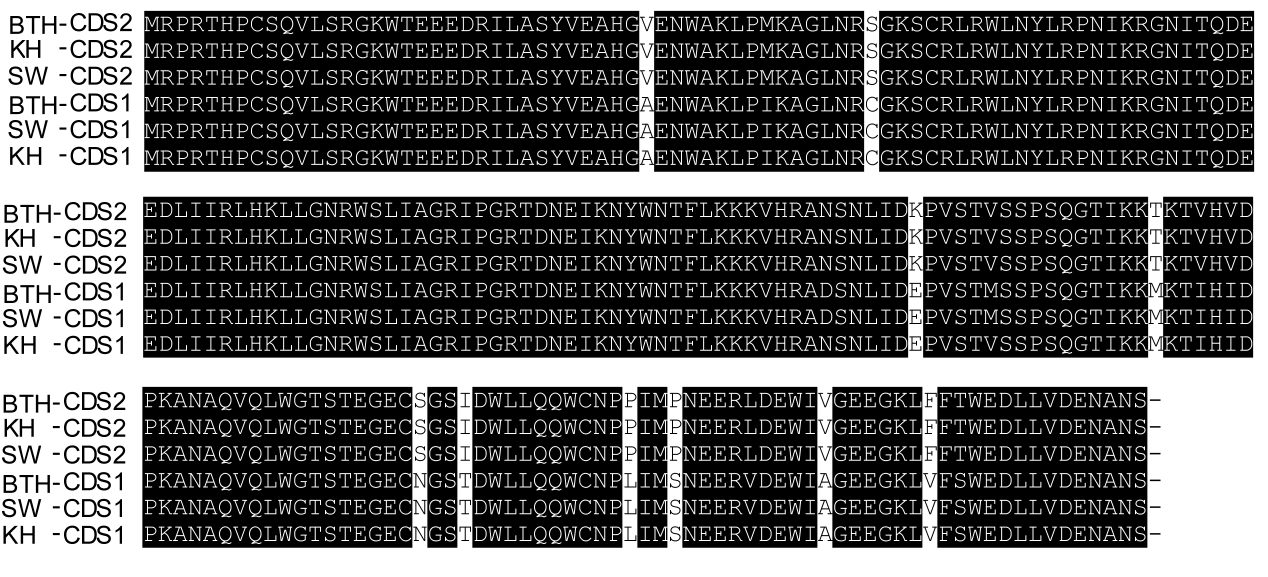


Figure S7. The coding sequence of AcMYB266 in ‘KH’, ‘BTH’, and ‘SW’ varieties


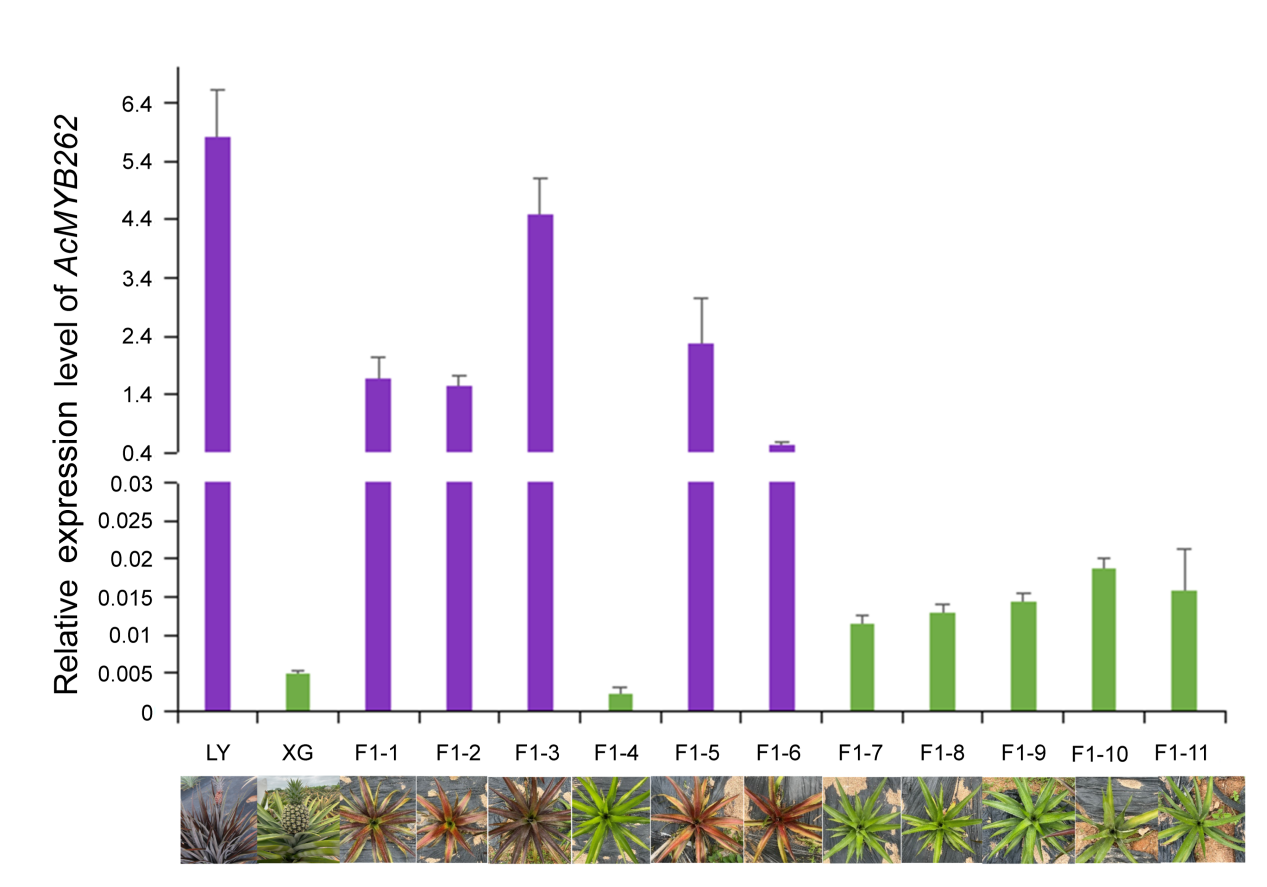


Figure S8. Expression patterns of *AcMYB262* in pineapple hybrid F_1_

The relative expression levels of *AcMYB262* in the F_1_ of ‘LY’ × ‘XG’.


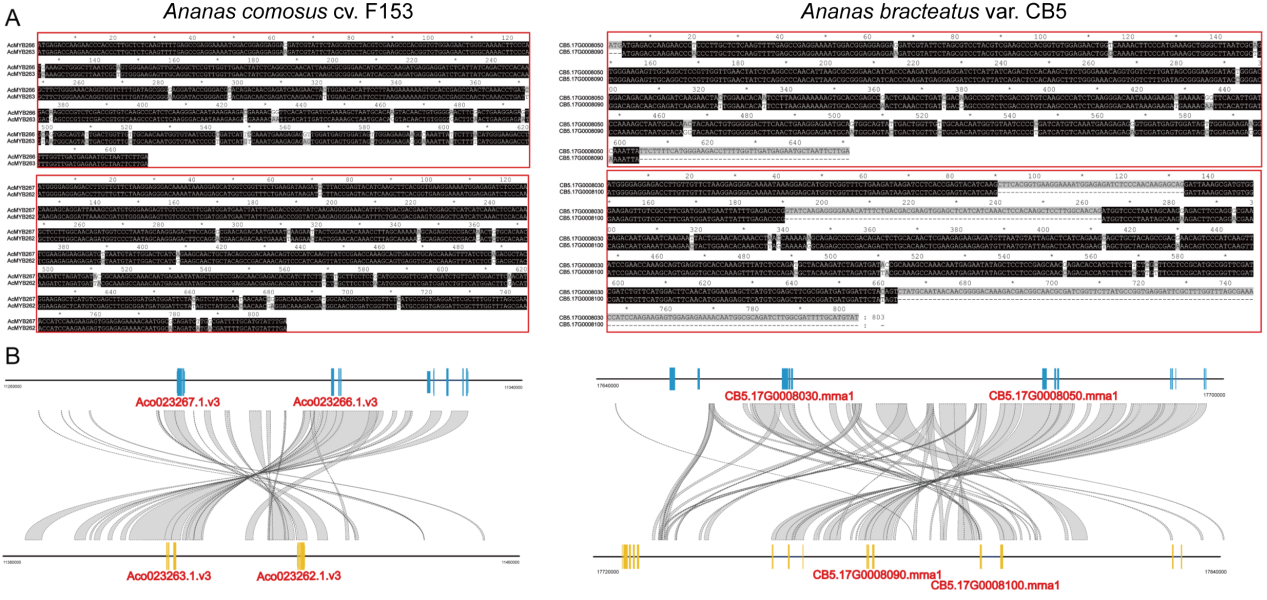


Figure S9. AcMYBs sequence analysis and collinearity analysis

1. Left: the alignment of AcMYB266 and AcMYB263, AcMYB267 and AcMYB262 nucleic acid sequences in ‘F153’, Right: the alignment of CB5.17G0008050 and CB5.17G0008090, CB5.17G0008030 and CB5.17G0008100 nucleic acid sequences in ‘CB5’; B. Collinearity analysis between AcMYBs and CB5-MYBs.


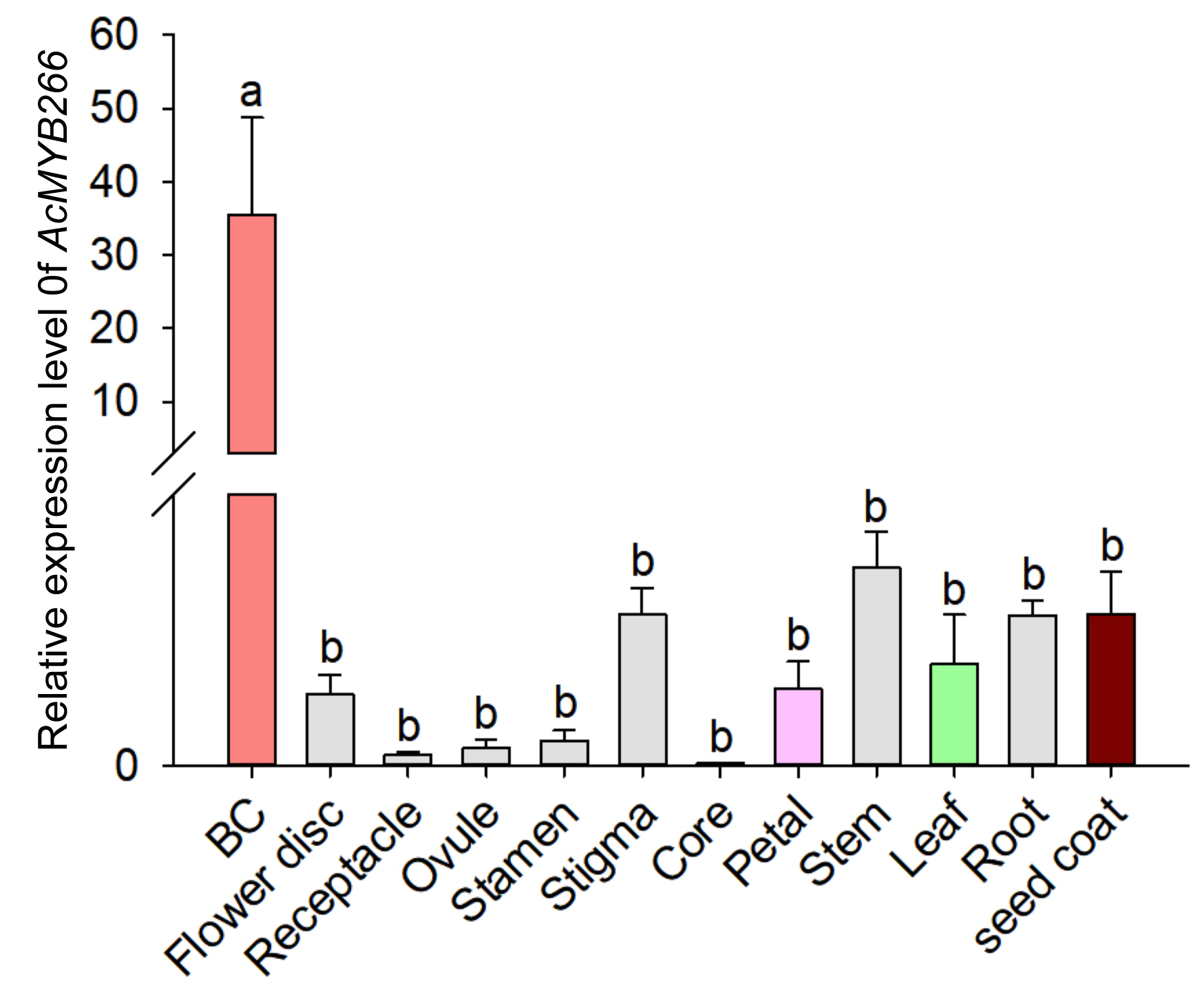


Figure S10. Expression pattern of *AcMYB266* in various tissues of ‘SW’

BC: the bracts and persistent calyx.

Table S1. Primer sequences required for this research experiment

| Primers | | sequences(5’-3’) |
| --- | --- | --- |
| NtActin | F | TCACAGAAGCTCCTCCTAATCCA |
|  | R | GAGGGAAAGAACAGCCTGAATG |
| AtActin | F | GCTCCTCTTAACCCAAAGGC |
|  | R | CACACCATCACCAGAATCCAGC |
| AcActin | F | CTGGCCTACGTGGCACTTGACTT |
|  | R | CACTTCTGGGCAGCGGAACCTTT |
| AcMYB266-CDS | F | ATGAGACCAAGAACCCACCCTT |
|  | R | TCAAGAATTAGCATTCTCATCAA |
| AcMYB266-pDONR | F | aaaaagcaggctccATGAGACCAAGAACCCACCCTT |
|  | R | AGAAAGCTGGGTTTCAAGAATTAGCATTCTCATCAA |
| AcMYB266  -TRV2 | F | aaggttaccgaattctctagaATGAGACCAAGAACCCACCCTT |
|  | R | cgtgagctcggtaccggatccTCAAGAATTAGCATTCTCATCAA |
| AcMYB266  -pGADT7 | F | gccatggaggccagtgaattcATGAGACCAAGAACCCACCCTT |
|  | R | attcatctgcagctcgagctcTCAAGAATTAGCATTCTCATCAA |
| AcbHLH901-pGBKT7 | F | atggccatggaggccgaattcATGGTTGCCCTTTCTCCAATAA |
|  | R | ggttatgctagttatgcggccgcTTAACTGTTTGAGAACACTC |
| AcCHSpro-pAbAi | F | cttgaattcgagctcggtaccAAGTCAGAGCCATCATCA |
|  | R | agcacatgcctcgaggtcgacGCACCACATCTAGTTTTCT |
| AcDFRpro-pAbAi | F | cttgaattcgagctcggtaccCGCTAAATCCTTAGTTCTCC |
|  | R | agcacatgcctcgaggtcgacCTCTCCCCCGATCTCTACAC |
| AcANSpro-pAbAi | F | cttgaattcgagctcggtaccGTGAGCCTCATTACAATAGCC |
|  | R | agcacatgcctcgaggtcgacATACACAATAATACACACTCTCC |
| AcUFGTpro-pAbAi | F | cttgaattcgagctcggtaccAGCGTTGCTACACTTCTT |
|  | R | agcacatgcctcgaggtcgacTGTTATAGTGTCTTCGAGTGGG |
| AcCHSpro-p0800-Luc | F | cttgaattcgagctcggtaccATTTGGATTATCGCTTGCTTTTAA |
|  | R | tgtttttggcgtcttccatggCATCGCCCTCGCTACTCTATCC |
| AcDFRpro-p0800-Luc | F | cttgaattcgagctcggtaccCGCTAAATCCTTAGTTCTCC |
|  | R | tgtttttggcgtcttccatggCTCTCCCCCGATCTCTACAC |
| AcANSpro-p0800-Luc | F | cttgaattcgagctcggtaccGTGAGCCTCATTACAATAGCC |
|  | R | tgtttttggcgtcttccatggATACACAATAATACACACTCTCC |
| AcUFGTpro-p0800-Luc | F | cttgaattcgagctcggtaccCCCAGGTACTAACTAGAAGAA |
|  | R | tgtttttggcgtcttccatggTTTAGGGGGGAGGGTTGCAA |
| AcMYB266 promoter | F | TGCTGTAATCAAGTCCCG |
|  | R | GGTGGGTTCTTGGTCTCAT |
